# Role of Na^+^/Ca^2+^ Exchanger (NCX) in Modulating Postovulatory Aging of Mouse and Rat Oocytes

**DOI:** 10.1371/journal.pone.0093446

**Published:** 2014-04-02

**Authors:** Chuan-Xin Zhang, Wei Cui, Min Zhang, Jie Zhang, Tian-Yang Wang, Jiang Zhu, Guang-Zhong Jiao, Jing-He Tan

**Affiliations:** College of Animal Science and Veterinary Medicine, Shandong Agricultural University, Tai-an City, P. R. China; Institute of Zoology, Chinese Academy of Sciences, China

## Abstract

We studied the role of the Na^+^/Ca^2+^ exchanger (NCX) in modulating oocyte postovulatory aging by observing changes in NCX contents and activities in aging mouse and rat oocytes. Whereas the NCX activity was measured by observing oocyte activation following culture with NCX inhibitor or activator, the NCX contents were determined by immunohistochemical quantification. Although NCX was active in freshly-ovulated rat oocytes recovered 13 h post hCG injection and in aged oocytes recovered 19 h post hCG in both species, it was not active in freshly-ovulated mouse oocytes. However, NCX became active when the freshly-ovulated mouse oocytes were activated with ethanol before culture. Measurement of cytoplasmic Ca^2+^ revealed Ca^2+^ increases always before NCX activation. Whereas levels of the reactive oxygen species (ROS) and the activation susceptibility increased, the density of NCX member 1 (NCX1) decreased significantly with oocyte aging in both species. While culture with H_2_O_2_ decreased the density of NCX1 significantly, culture with NaCl supplementation sustained the NCX1 density in mouse oocytes. It was concluded that (a) the NCX activity was involved in the modulation of oocyte aging and spontaneous activation; (b) ROS and Na^+^ regulated the NCX activity in aging oocytes by altering its density as well as functioning; and (c) cytoplasmic Ca^2+^ elevation was essential for NCX activation in the oocyte.

## Introduction

Mammalian oocytes are arrested at the meiotic metaphase II (MII) stage following ovulation. If not fertilized in time, the ovulated oocytes undergo a time-dependent process of aging [1], [2]. In vitro culture of matured oocytes also leads to oocyte aging [3]–[6]. The postovulatory oocyte aging has marked detrimental effects on embryo development [5], [7]–[9] and offspring [10], [11]. Furthermore, the use of aged oocytes resulted in significant decrease in embryonic development following in vitro fertilization, intracytoplasmic sperm injection [12] or nuclear transfer [13]–[15]. Thus, studies on the mechanisms and control of oocyte aging are important for both normal and assisted reproduction. However, the mechanisms for oocyte aging are not fully clear.

In spite of intensive efforts, many studies have failed to obtain rat offspring by nuclear transfer of somatic cells [16]–[20]. Unlike oocytes from other mammals, the rat oocytes undergo spontaneous activation soon after collection from the oviduct [21], [22]. Rat somatic cell nuclei introduced into enucleated rat oocytes do not show premature chromosome condensation [17] and might not be properly reprogrammed due to oocyte spontaneous activation during nuclear transfer manipulation [23]. Inhibiting oocyte spontaneous activation is thus of great importance for successful rat cloning. However, the mechanisms causing the spontaneous activation of rat oocytes are not fully understood.

Mammalian oocytes are activated by intracellular Ca^2+^ oscillations both at fertilization [24], [25] and at parthenogenetic activation [26]–[30]. Furthermore, our recent study observed Ca^2+^ increases in rat oocytes during spontaneous activation [31]. There are two major mechanisms responsible for Ca^2+^ extrusion across the plasma membrane: the Ca^2+^–ATPase and Na^+^/Ca^2+^ exchange [32], [33]. The Na^+^/Ca^2+^ exchange has been observed in immature and mature mouse [34], [35], porcine [36] and rat [37] oocytes. However, although our recent study indicated a role for Na^+^/Ca^2+^ exchange in the spontaneous activation of rat oocytes [37], systematic studies are lacking on roles of the Na^+^/Ca^2+^ exchanger (NCX) activity in controlling postovulatory oocyte aging of different species. Furthermore, in previous studies the NCX activity was usually determined by functioning assays (by measuring Ca^2+^ flux, for example), with little attention paid to the dynamic changes in NCX contents.

Post-ovulatory aging of oocytes is associated with significant decreases in cytoplasmic reduced glutathione [38], [39] indicating a loss of cellular protection against oxidative stress [40]. It is known that oocyte aging alters the regulation of the intracellular calcium concentration, thus affecting Ca^2+^ oscillations in fertilized oocytes [41]. However, although reactive oxygen species (ROS) are known to significantly perturb Ca^2+^ homeostasis mainly through direct effects on the machinery involved in intracellular Ca^2+^ storage [42], [43], the effect of oxidative stress on the NCX activity has not been observed in aging oocytes.

The objective of the current study was to determine the role of NCX in modulating oocyte postovulatory aging by observing changes in NCX contents and activities in aging mouse and rat oocytes and to study the effect of oxidative stress on the NCX activity during oocyte aging.

## Results

### The NCX is active in rat but not in mouse freshly-ovulated oocytes

To test the NCX activity in freshly-ovulated oocytes, mouse and rat oocytes collected 13 h post hCG injection were cultured for 6 h in CZB and mR1ECM medium, respectively, with different supplements to modulate the NCX activity. At the end of culture, while rat oocytes were observed for spontaneous activation immediately, mouse oocytes were treated with 10% ethanol for 10 min, cultured in CZB without supplements and observed for activation 6 h later. Whereas percentages of the spontaneously activated rat oocytes increased significantly, percentages of the ethanol activated mouse oocytes did not change after treatment with different concentrations of bepridil (Table 1). Although NaCl supplementation inhibited spontaneous activation of rat oocytes, ethanol activation of mouse oocytes increased significantly with increasing concentrations of NaCl (Table 2). Results suggest that NCX is active in freshly-ovulated rat oocytes but not in freshly-ovulated mouse oocytes.

**Table 1:** Activation rates after mouse or rat oocytes collected 13(mouse) or mR1ECM (rat) medium supplemented with or without bepridil.

Bepridil (μM)	Mouse		Rat	
	Oocytes observed	% Activated oocytes	Oocytes observed	% Activated oocytes
0	139	38.2±2.4^a^	167	38.5±3.7^a^
12.5	134	31.3±3.4^a^	-	-
25	136	36.3±2.6^a^	-	-
37.5	131	33.1±2.2^a^	-	-
50	130	36.6±1.6^a^	142	60.4±3.0^b^

a,b: Values with a common letter in their superscripts do not differ (P>0.05) in the same column. While rat oocytes were examined for spontaneous activation immediately after culture, mouse oocytes were treated with 10% ethanol for 10 min after culture and observed for activation 6 h later.

**Table 2:** Activation rates after mouse or rat oocytes collected 13(mouse) or mR1ECM (rat) medium supplemented with different concentrations of NaCl.

NaCl (mM)	Mouse		Rat	
	Oocytes cultured	% Activated oocytes	Oocytes cultured	% Activated oocytes
0	125	35.5±2.2^a^	118	36.7±4.2^a^
25	122	58.3±2.1^b^	-	-
50	115	66.3±3.5^bc^	106	6.2±1.6^b^
75	123	72.6±3.3^c^	-	-

a–c: Values with a common letter in their superscripts do not differ (P>0.05) in the same column. While rat oocytes were examined for spontaneous activation immediately after culture, mouse oocytes were treated with 10% ethanol for 10 min after culture and observed for activation 6 h later.

### NCX is active in aged oocytes in both mice and rats

To test the NCX activity in aged oocytes, mouse and rat oocytes collected 19 h post hCG injection were cultured for 6 h with supplementation of 50 μM bepridil or different concentrations of NaCl. While rat oocytes were examined for spontaneous activation immediately after culture, mouse oocytes were treated with 5% ethanol for 10 min after culture and observed for activation 6 h later. Activation rates increased significantly in both mouse and rat oocytes after culture with 50 μM bepridil (Table 3). Supplementation with 75 mM and 50 mM NaCl significantly inhibited activation of mouse and rat oocytes, respectively (Table 4). Results suggest that the NCX is active in aged oocytes of both species.

**Table 3:** Activation rates after mouse or rat oocytes collected 19(mouse) or mR1ECM (rat) medium supplemented with or without bepridil.

Bepridil (μM)	Mouse		Rat	
	Oocytes observed	% Activated oocytes	Oocytes observed	% Activated oocytes
0	113	40.5±1.4^a^	104	70.3±1.8^a^
50	112	56.8±1.6^b^	108	98.0±2.0^b^

a,b: Values with a common letter in their superscripts do not differ (P>0.05) in the same column. While rat oocytes were examined for spontaneous activation immediately after culture, mouse oocytes were treated with 5% ethanol for 10 min after culture and observed for activation 6 h later.

**Table 4:** Activation rates after mouse or rat oocytes collected 19(mouse) or mRECM (rat) medium supplemented with different concentrations of NaCl.

NaCl (mM)	Mouse		Rat	
	Oocytes observed	% Oocytes activated	Oocytes observed	% Oocytes activated
0	131	45.2±4.3^a^	123	69.1±2.3^a^
25	130	44.4±3.7^a^	-	-
50	134	49.3±2.7^a^	130	21.1±1.8^b^
75	135	21.9±4.7^b^	-	-

a–b: Values with a common letter in their superscripts do not differ (P>0.05) in the same column. While rat oocytes were examined for spontaneous activation immediately after culture, mouse oocytes were treated with 5% ethanol for 10 min after culture and observed for activation 6 h later.

### NCX became active when freshly-ovulated mouse oocytes were activated with ethanol

The objective of this experiment was to find out (a) why NCX was not active in freshly-ovulated mouse oocytes while it was in freshly-ovulated rat oocytes and (b) whether the NCX could be activated by activating mouse oocytes. Mouse oocytes collected 13 h post hCG were first treated for activation with 10% ethanol for 10 min and then cultured for 6 h in CZB medium supplemented with different concentrations of ouabain or NaCl. Ouabain was used in place of bepridil because many mouse oocytes lysed when treated with bepridil following ethanol activation treatment. At the end of culture, oocytes were examined for activation. Activation rates increased with increasing concentrations of ouabain but decreased with increasing NaCl concentrations significantly (Table 5). Results suggested that NCX could be activated only after the freshly-ovulated mouse oocytes were pre-activated to some extent. Since 40% of the freshly-ovulated rat oocytes underwent spontaneous activation after culture, it was suggested that the freshly-ovulated rat oocytes are prone to spontaneous activation because they were halfway activated soon after the release from oviducts.

**Table 5:** Activation rates after mouse oocytes collected 13% ethanol for 10 min and cultured for 6 h in CZB medium supplemented with different concentrations of ouabain or NaCl.

Ouabain			NaCl		
mM	Oocytes observed	% Oocytes activated	mM	Oocytes observed	% Oocytes activated
0	116	31.1±4.1^a^	0	121	31.8±2.9^a^
0.25	121	48.8±1.7^b^	25	111	22.8±5.2^a^
0.5	128	61±4.2^c^	50	115	5.24±1.6^b^
1	108	76.6±2.9^d^	75	110	0.0±0.0^b^
2	114	73.1±1.7^d^	-	-	-

a–d: Values with a common letter in their superscripts do not differ (P>0.05) in the same column. Ouabain was used in place of bepridil because many mouse oocytes lysed when treated with bepridil following ethanol activation treatment.

### A cytoplasmic Ca^2+^ rise is a prerequisite for NCX activation

**Figure 1:**
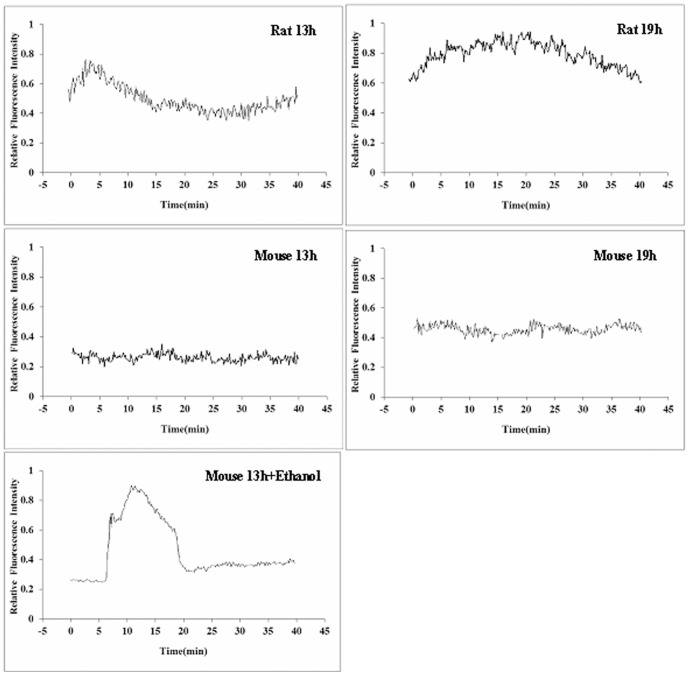
Calcium measurements in rat and mouse oocytes collected at 13 h or 19 h after hCG injection. Calcium was also measured after mouse oocytes recovered at 13% ethanol for 10 min (Mouse 13h+Ethanol). Oocytes recovered at different times were cultured for 40 min in M2 (mouse) or HR1 (rat) and the calcium concentrations were measured during the culture. Each treatment was repeated three times, with each replicate containing about 20 oocytes.

Because the above results suggested that NCX could be activated only after oocytes were pre-activated to some extent and because Ca^2+^ rises were reported at oocyte activation, we proposed that Ca^2+^ increases might be essential for NCX activation. To test this hypothesis, cytoplasmic concentrations of Ca^2+^ were measured in mouse and rat oocytes recovered at different times after hCG injection. Whereas rat oocytes recovered either 13 or 19 h after hCG injection showed marked Ca^2+^ rises, freshly-ovulated mouse oocytes recovered 13 h post hCG did not show any Ca^2+^ increase (Fig. 1). Although mouse oocytes collected 19 h after hCG did not show marked Ca^2+^ oscillations, their cytoplasmic Ca^2+^ concentrations were much higher than those observed in the freshly-ovulated mouse oocytes. Furthermore, the freshly-ovulated mouse oocytes showed marked Ca^2+^ oscillations following treatment with ethanol. In summary, Ca^2+^ rises were obvious always in those oocytes in which marked NCX activities had been observed in the above experiments. Thus, the results confirmed that NCX could be activated only in those oocytes that had shown a Ca^2+^ elevation.

### Changes in ROS levels, the activation susceptibility and the density of NCX member 1 (NCX1) during oocyte aging

**Figure 2:**
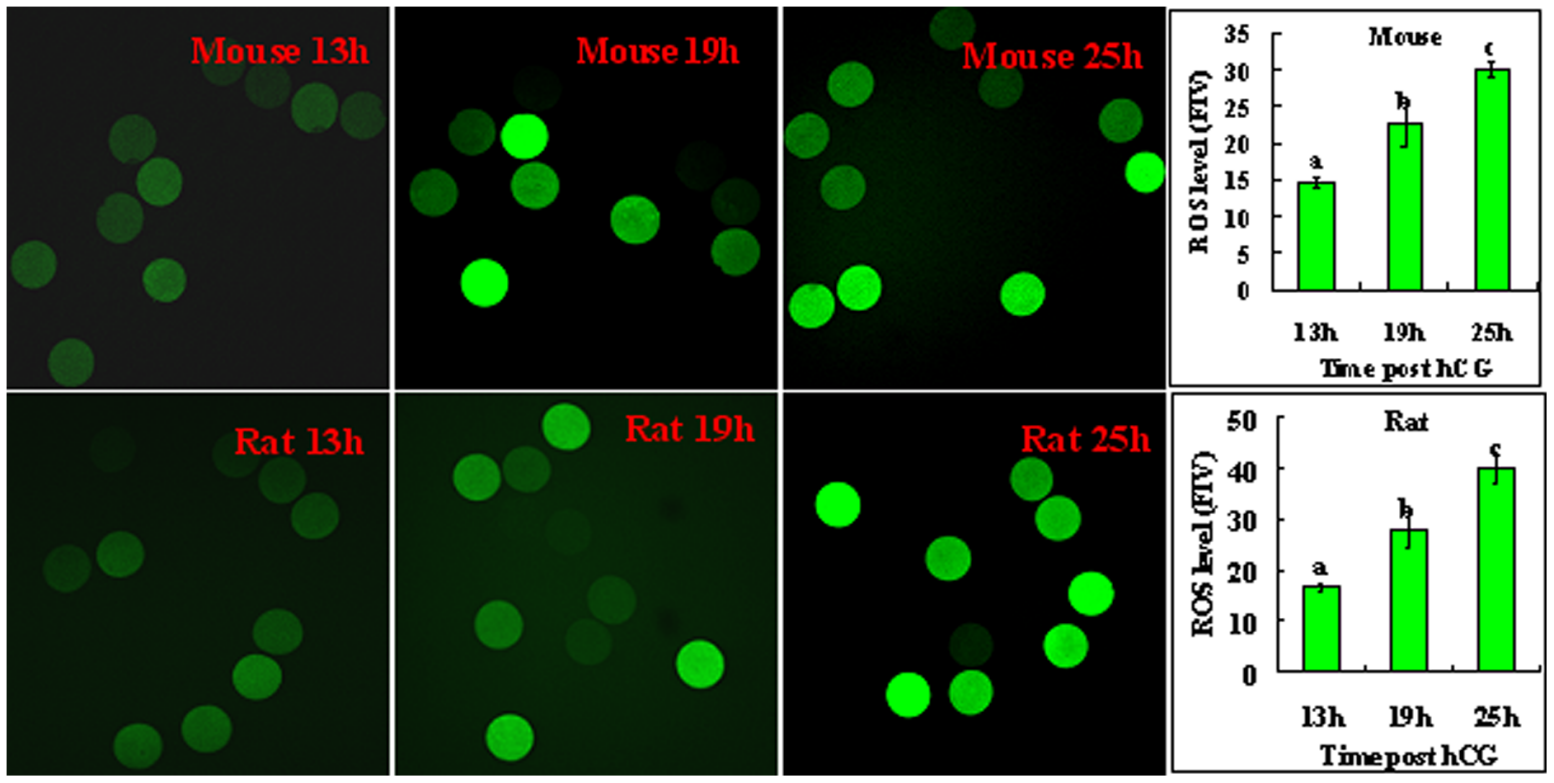
Levels of ROS (fluorescence intensity) in mouse and rat oocytes recovered at different times after hCG injection. The images show mouse or rat oocytes collected at 13(fluorescence intensity value, FIV) in oocytes recovered at different times after hCG injection. Each treatment was repeated 3–4 times with each replicate containing about 30 oocytes. a,b,c: Values without a common letter in their bars differ (P<0.05) within graphs.

Because oxidative stress had been reported in aging oocytes, its effect on the NCX density and activation susceptibility was observed in aging oocytes. The intra-oocyte ROS levels, rates of activation, and the NCX1 density were measured in oocytes recovered at different times after hCG injection. Both the ethanol activation rates of mouse oocytes and the spontaneous activation rates of rat oocytes increased significantly with increasing time after hCG injection (Table 6). Although the ROS level in freshly-ovulated oocytes was low, it increased dramatically with oocyte aging in both species (Fig. 2).

**Table 6:** Activation rates of mouse or rat oocytes collected at different times post hCG injection.

Time (h) post hCG	Mouse		Rat	
	Oocytes observed	% Activated oocytes	Oocytes observed	% Activated oocytes
13	117	4.4±1.2^a^	137	38.5±3.7^a^
19	114	46.1±3.3^b^	122	70.3±2.5^b^
25	113	95.6±2.9^c^	134	82.3±2.3^c^

a–c: Values with a common letter in their superscripts do not differ (P>0.05) in the same column. While rat oocytes were examined for spontaneous activation after culture for 6 h in mR1ECM, mouse oocytes were treated with 5% ethanol for 10 min immediately after collection and observed for activation 6 h later.

**Figure 3:**
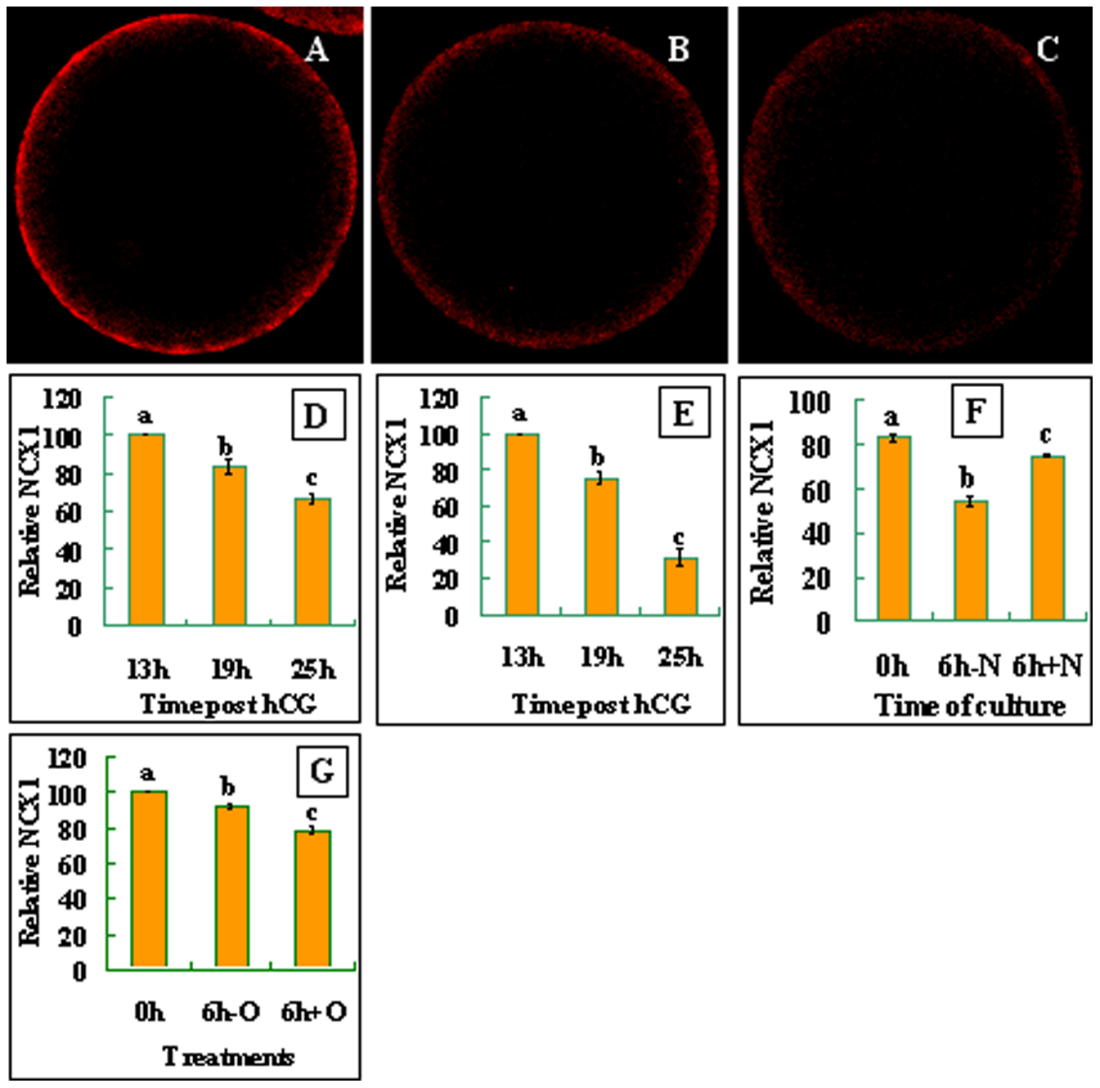
Distribution and quantification of NCX1 in mouse and rat oocytes. The micrographs are laser confocal images (equatorial sections) of oocytes with NCX1 pseudo-colored red. They show the cortical distribution of NCX1 in newly-ovulated mouse oocytes (A) or oocytes cultured for 6 h in CZB medium without (B) or with (C) H_2_O_2_ addition. Graphs D and E show NCX1 quantification, respectively, in mouse and rat oocytes recovered at different times post hCG injection. Graph F shows NCX1 quantification after mouse oocytes recovered 19 h post hCG were cultured for 6 h with (+N) or without (-N) 75 mM NaCl supplementation. Graph G shows NCX1 quantification in newly-ovulated mouse oocytes (0h), or oocytes cultured for 6 h in CZB medium with (6h+O) or without (6h-O) H_2_O_2_ addition. Each treatment was repeated four times, with each replicate containing 7–10 oocytes. a-c: Values without a common letter above their bars differ (P<0.05).

When observed under a laser confocal microscope after labeling with NCX1 antibodies, NCX1 was localized in the egg cortex, suggesting its localization in the plasma membrane of oocytes (Fig. 3A, B and C). Quantification analysis showed that the density of NCX1 decreased significantly with aging of both mouse (Fig. 3D) and rat (Fig. 3E) oocytes. Together, the results suggested that whereas the ROS levels and the activation susceptibility increased, the density of NCX1 decreased significantly with oocyte aging in both species.

### Aged oocytes showed lower NCX activities due to their decreased NCX densities

To determine the correlation between the density and the activity of NCX, NCX activities in rat oocytes collected at different times after ovulation were estimated by calculating the times of activation rates between oocytes cultured without and with 50 mM NaCl supplementation. Results showed that the times of activation rates decreased from 6 (37/6.2, Table 2) at 13 h to 3.3 (69/21, Table 4) at 19 h and to 1.6 (83/52, data not shown) at 25 h post hCG injection. Together with the result that the density of NCX1 decreased significantly with oocyte aging (Fig. 3D and E), it was suggested that the decreased NCX activity in aged oocytes was resulted from their reduced NCX densities. To further confirm the correlation between the density and the activity of NCX, the NCX1 density was compared among mouse oocytes that showed different NCX activities following culture with or without NaCl supplementation. When oocytes recovered 19 h post hCG injection were cultured for 6 h without NaCl, the level of NCX1 decreased significantly (Fig 3F). When the oocytes were cultured with NaCl, however, the decrease in the NCX1 density was significantly inhibited.

### Increasing the ROS level artificially decreased the density of NCX1 significantly in mouse oocytes

To confirm the effect of oxidative stress on the density of NCX during oocyte aging, mouse oocytes recovered 13 h post hCG were cultured for 6 h in the presence or absence of 100 μM H_2_O_2_ before examination for NCX1 densities. Results showed that the NCX1 density was significantly lower in oocytes cultured with than without H_2_O_2_ (Fig. 3G).

## Discussion

In the present study, whereas NaCl was used to increase the NCX activity, bepridil or ouabain was used to inhibit it. Bepridil was used for rat and mouse oocytes when no ethanol activation was involved, while ouabain was used for mouse oocytes after ethanol activation treatment because many mouse oocytes lysed when treated with bepridil following ethanol treatment. It is known that the NCX uses the electrochemical gradient of Na^+^ across the plasma membrane to exchange three Na^+^ ions into the cell for the extrusion of one Ca^2+^ ion [44]–[47]. Thus, maneuvers that increase the extracellular Na^+^ will activate the NCX and promote Ca^2+^ efflux [34], [48], [49]. Bepridil is a commonly used NCX inhibitor in different cell types, including oocytes [34], [36], [50], [51]. Ouabain is a specific inhibitor of Na^+^/K^+^-ATPase (NKA). As the extracellular concentrations of Na^+^ and Ca^2+^ remain close to constant, NCX activity is largely under the influence of the intracellular Ca^2+^ and Na^+^ concentrations [52]. The intracellular Na^+^ is mainly controlled by NKA [53], down regulation of which caused attenuated control of NCX activity, reducing its capability to extrude Ca^2+^ from cells [54], [55].

The present results showed that although NCX was active in aged oocytes recovered 19 h post hCG injection in both mice and rats, and in freshly-ovulated rat oocytes as well, it was not active in freshly-ovulated mouse oocytes. However, NCX was activated when the freshly-ovulated mouse oocytes were activated with ethanol. Our further observations on different oocytes indicated that there were always cytoplasmic Ca^2+^ increases in oocytes in which the NCX was proven active. Thus, whereas the freshly-ovulated mouse oocytes showed neither NCX activities nor Ca^2+^ increases when observed immediately after recovery, they displayed marked cytoplasmic Ca^2+^ rises as well as NCX activities when observed after pre-culture ethanol activation. We therefore concluded that a cytoplasmic Ca^2+^ elevation was essential for NCX activation in the oocyte. There are two major mechanisms to extrude Ca^2+^ across the plasma membrane: the Ca^2+^–ATPase and the Na^+^/Ca^2+^ exchanger [32], [33]. However, the threshold intracellular Ca^2+^ for the activation of the two transporters is different; whereas the Ca^2+^–ATPase needs a low threshold level, the Na^+^/Ca^2+^ exchanger requires a high threshold level of cytoplasmic Ca^2+^ to activate [57]. For example, whereas in resting cells, where the cytoplasmic Ca^2+^ concentration is approximately 100 nM [56], the Ca^2+^–ATPase plays an important role in maintaining resting cytoplasmic Ca^2+^, NCX is activated to transport Ca^2+^ when the cytoplasmic concentration of Ca^2+^ increased to the micromolar range [57].

**Figure 4:**
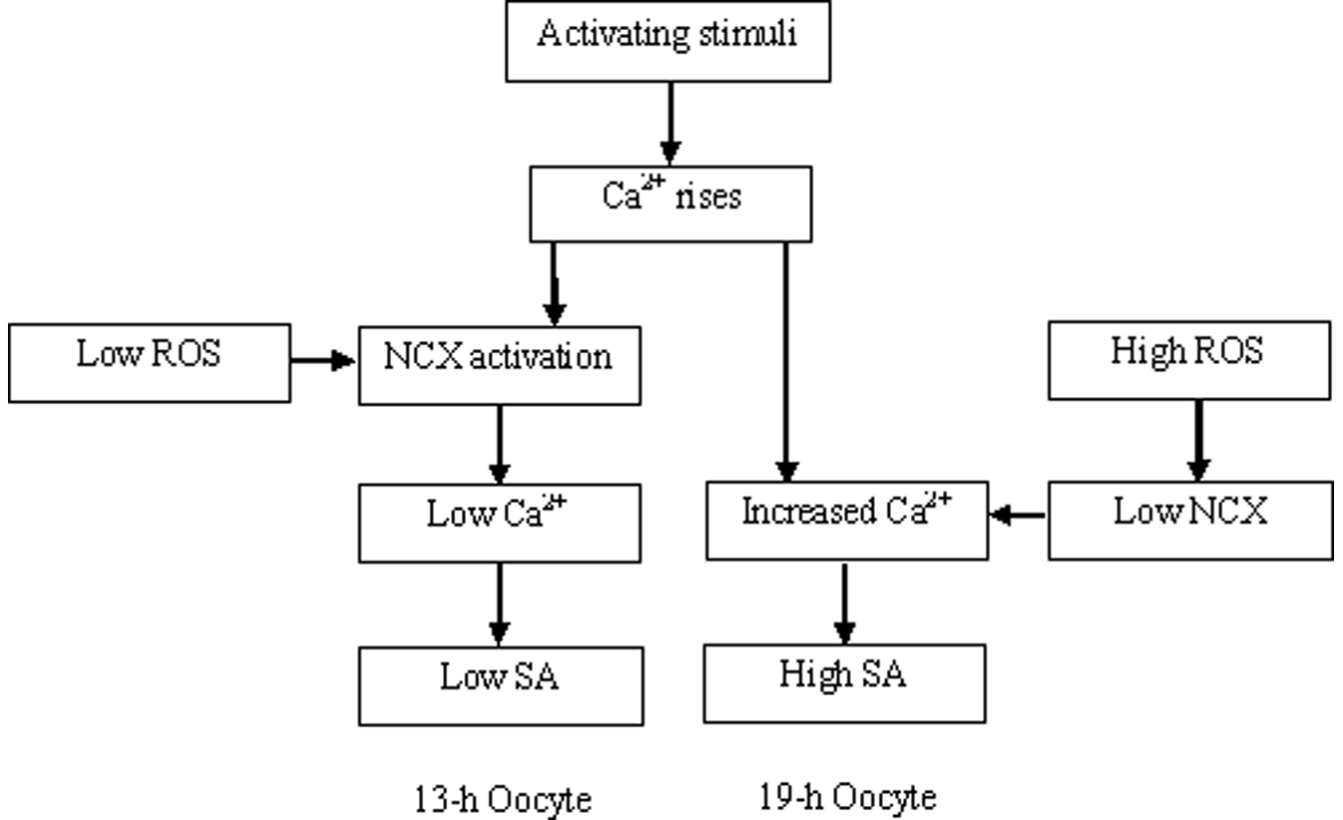
Possible pathways leading spontaneous activation (SA) in freshly ovulated (13 h) and aged (19 h) rat oocytes.

Both the present study and our previous studies [31], [37] demonstrated that in the Sprague-Dawley rats, whereas many oocytes recovered 19 h post hCG injection underwent spontaneous activation during in vitro culture, spontaneous activation was less frequently observed in oocytes collected 13 h after hCG. To explore the mechanisms causing the difference in activation susceptibility between newly-ovulated and aged rat oocytes, the present results indicated that in the 13-h oocytes, an unknown activating stimulus (maybe the manipulation for their release from the oviduct) induced mild Ca^2+^ rises, which activated NCX under a low ROS level (Fig. 4). The activated NCX then transported Ca^2+^ out of the oocyte, leading to a low level of spontaneous activation in this group of oocytes. In the 19-h oocytes, however, because the high level of ROS inhibited NCX, the cytoplasmic Ca^2+^ rises induced by the unknown stimulus continued to enlarge leading to spontaneous activation in many oocytes. An increase in ROS has been observed with the aging of oocytes [40]. Many studies reveal that ROS inactivates NCX, leading to a rise in cytoplasmic Ca^2+^ and subsequent cell dysfunction [57]. For instance, in dialyzed squid axons, oxidative stress inhibits NCX by impairing the intracellular Ca^2+^-regulatory site [58]. In cardiac myocytes exposed to ischemia/reperfusion, which produces a burst of oxygen radicals, the NCX can operate in the reverse mode causing Ca^2+^ influx into the cytoplasm [59]. In addition, the present results provided further evidence that the extra-cellular molecules regulate the NCX activity by changing its density. Thus, while ROS increased oocyte activation susceptibility by decreasing the NCX density, Na^+^ supplementation increased the NCX activity by maintaining its density in aging oocytes.

Although it is known that freshly-ovulated rat oocytes underwent spontaneous activation immediately after their release from the oviduct while freshly-ovulated mouse oocytes did not, the mechanisms are not clear. The present results demonstrated that whereas 30–40% of the freshly-ovulated rat oocytes underwent spontaneous activation after in vitro culture for 6 h, none of the freshly-ovulated mouse oocytes showed spontaneous activation (data not shown). Similarly, whereas most of the newly-ovulated rat oocytes showed cytoplasmic Ca^2+^ rises immediately after collection, no Ca^2+^ increase was observed in any of the newly-ovulated mouse oocytes. However, over 30% of the freshly-ovulated mouse oocytes were activated and marked cytoplasmic Ca^2+^ rises were observed after treatment with 10% ethanol for 10 min. Thus, the newly-ovulated rat oocytes differ from the newly-ovulated mouse oocytes in activation susceptibility because the former showed initial increases in cytoplasmic Ca^2+^ while the latter did not. Furthermore, whereas the rat oocytes recovered at 13 h and 19 h post hCG and the ethanol-activated mouse oocytes showed Ca^2+^ oscillations, mouse oocytes recovered 19 h after hCG injection showed only an increase in Ca^2+^ concentration but not typical Ca^2+^ oscillations. This suggests that whereas a threshold level of cytoplasmic Ca^2+^ is enough for NCX activation, spontaneous activation of oocytes requires Ca^2+^ oscillations, because mouse oocytes recovered 19 h after hCG injection did not undergo spontaneous activation while the rat oocytes recovered at 13 h or 19 h post hCG did.

Many studies have shown that oocyte activation is associated with intracellular Ca^2+^ oscillations [24]–[30]. Researches on the necessary concentration of, and exposure time to, ethanol to induce activation of newly-ovulated mouse oocytes indicated that using a lower concentration for a shorter time was obviously without effect. Our data (not shown) indicated that the freshly-ovulated mouse oocytes were not activated at all when exposed to a weaker stimulus (5% ethanol for 5 min). This suggests that the freshly-ovulated rat oocytes are prone to spontaneous activation because the release from the oviduct acts as a strong activating stimulus (like treatment of the freshly-ovulated mouse oocytes with 10% ethanol for 10 min) that increases their activation susceptibility via inducing Ca^2+^ oscillations.

In summary, we have studied the role of the NCX activity in modulating postovulatory aging of mouse and rat oocytes. The results showed that (a) the NCX activity was involved in the modulation of oocyte aging and spontaneous activation; (b) whereas ROS increased oocyte activation susceptibility by decreasing the NCX density, Na^+^ inhibited oocyte increase of activation susceptibility by maintaining the NCX density, suggesting that extra-cellular molecules regulated the NCX activity of aging oocytes by modifying its density as well as functioning; and (c) a cytoplasmic Ca^2+^ elevation was essential for NCX activation in the oocyte. The data are important for our understanding of not only the mechanisms for aging and spontaneous activation of oocytes but also the cellular mechanisms regulating the NCX activity.

## Materials and Methods

### Ethics Statement

Mouse car and use were conducted exactly in accordance with the guidelines and approved by the Animal Research Committee of the Shandong Agricultural University, P. R. China (Permit number: 20010510). According to the guidelines of the committee, the animal handling staff (including each post-doc, doctoral or masters student) must be trained before using animals. Mice must be housed in a temperature-controlled room with proper darkness-light cycles, fed with a regular diet, and maintained under the care of the Experimental Animal Center, Shandong Agricultural University College of Animal Science and Vet Medicine. In the present study, mice were sacrificed by cervical dislocation. The only procedure performed on the dead animals was the collection of oocytes from the ovaries.

Chemicals and reagents used in this study were purchased from Sigma Chemical Co. (St. Louis, MO, USA) unless otherwise specified.

### Oocyte recovery

Mice of the Kunming breed and rats of the Sprague-Dawley breed were kept in an air-conditioned room with 14 h/10 h light-dark cycles, the darkness starting from 8 pm. The animals were handled by the rules stipulated by the Animal Care and Use Committee of Shandong Agricultural University. Female mice (8–10 wk after birth) and rats (23–26 days after birth) were induced to superovulate by i.p. injection of equine chorionic gonadotropin (eCG) (10 IU for mice, 15 IU for rats), followed 48 h later by injection of human chorionic gonadotropin (hCG) (10 IU for mice, 15 IU for rats). Both eCG and hCG used in this study were from Ningbo Hormone Product Co., Ltd. The superovulated mice or rats were sacrificed at different times after hCG injection and the oviductal ampullae were broken to release oocytes. After dispersed and washed three times in M2 medium, the oocytes were denuded of cumulus cells by pipetting with a thin pipette in a drop of M2 containing 0.1% hyaluronidase.

### Oocyte aging in vitro

For in vitro aging, mouse or rat oocytes recovered at different times after hCG were cultured for 6 h in the aging medium supplemented with different substances. The aging medium used for mouse oocytes was a modified Chatot-Ziomek-Bavister (CZB) medium [60] while that for rat oocytes was the rat 1-cell embryo culture medium (mR1ECM) [61]. To prepare stock solutions, bepridil and ouabain were dissolved in DMSO at 50 mM and 400 mM, respectively. The stock solutions were stored in aliquots at −20°C and diluted to desired concentrations with CZB or mR1ECM immediately before use. H_2_O_2_ were diluted to 100 μM with CZB immediately before use. The aging culture was conducted in wells (20–35 oocytes per well) of a 96-well culture plate containing 200 μl of aging medium covered with mineral oil at 37°C under 5% CO_2_ in humidified air.

### Assessment of oocyte activation

Whereas rat oocytes were assessed for spontaneous activation immediately after aging culture, mouse oocytes were treated with ethanol to induce activation. For ethanol activation, mouse oocytes were first treated with ethanol in M2 medium for 10 min at room temperature, then washed three times and cultured in the CZB medium for 6 h. Whereas oocytes recovered 13 h post hCG were treated with 10% ethanol, oocytes collected 19 h post hCG were activated with 5% ethanol. This was done because whereas the freshly-ovulated mouse oocytes were not activated well with 5% ethanol, almost all the aged oocytes were activated with 10% ethanol regardless of treatments. At the end of culture, oocytes were observed under a microscope for activation. Only those oocytes that had one or two pronuclei or two cells each having a nucleus were considered activated. To observe spontaneous activation, rat oocytes were fixed with 3.7% paraformaldehyde in M2 for 30 min at room temperature before being stained with 10 μg/ml Hoechst 33342 and mounted on glass slides. The state of chromosomes was observed under an epifluorescence microscope (Leica DMLB) and was classified into two types. Oocytes with chromosomes compacted at the metaphase plate were considered to be at the metaphase II (MII) stage, whereas oocytes with chromosomes dispersed in the cytoplasm were classified as activated. Dispersed chromosomes in the cytoplasm, rather than the formation of pronuclei, were used as an indication of oocyte activation because the spontaneously activated rat oocytes are often arrested in metaphase III instead of forming pronuclei [31], [37].

### Ca^2+^ measurement

Intracellular Ca^2+^ was measured using the Ca^2+^-sensitive dye fluo-3. For loading, oocytes were incubated for 20 min at 37°C with 30 μM of the acetoxymethyl (AM) form of the dye made up in CZB (mouse) or mR1ECM (rat) with 0.02% pluronic F-127. After loading, mouse and rat oocytes were washed with and placed in a 22.5 μl drops of M2 and Hepes-buffered mR1ECM (HR1), respectively, under paraffin oil in a Fluoro dish (World Precision Instruments, Inc.) with its base coated with phytoagglutinin. The dish was transferred to a heated stage (37°C) of a Leica laser-scanning confocal microscope (TCS SP2; Leica Microsystems). In order to detect the mouse oocytes reaction to ethanol, 2.5 μl of absolute ethanol was injected into the M2 drop to initiate ethanol stimulation. The 10 min ethanol stimulation was terminated by diluting the drop with 1000 μl M2. Oocytes were observed using a 10× objective. An argon laser was used for excitation at 488 nm and signals emitted at 505–540 nm were collected for 40 min by the laser scanning confocal imaging system. Traces of Ca^2+^ oscillations were plotted using SigmaPlot 2000 software.

### Immunofluorescence microscopy

All the procedures were conducted at room temperature unless otherwise specified. Oocytes were washed 3 times in M2 between treatments. Oocytes were (i) fixed with 3.7% paraformaldehyde in PHEM buffer (60 mM Pipes, 25 mM Hepes, 10 mM EGTA and 4 mM MgSO_4_, pH 7.0) for at least 30 min, followed by treatment with 0.25% protease in M2 for 1 to 2 seconds to remove zona pellucida; (ii) permeabilized with 0.1% Triton X-100 in PHEM for 5 min; (iii) blocked in PHEM containing 3% BSA for 1 h; (iv) incubated overnight with mouse monoclonal anti-NCX1 (IgM, 1∶400, Abcam, Cambridge, MA) in 3% BSA in M2 at 4°C; (v) incubated for 1 h with Cy3-conjugated goat-anti-mouse IgM (1∶800, Jackson ImmunoResearch) in 3% BSA in M2.

The stained oocytes were mounted on glass slides and observed with a Leica laser scanning confocal microscope (TCS SP2). Helium/neon (He/Ne; 543 nm) lasers were used to excite Cy3, fluorescence was detected with bandpass emission filter (560–605 nm), and the captured signals were recorded as red. The relative content of NCX1 was quantified by measuring fluorescence intensities. For each experimental series, all high-resolution z-stack images were acquired with identical settings. The relative intensities were measured on the raw images using Image-Pro Plus software (Media Cybernetics Inc., Silver Spring, MD) under fixed thresholds across all slides.

### Assay for intraoocyte reactive oxygen species (ROS)

In order to quantify ROS in individual oocytes, intraoocyte H_2_O_2_ levels were measured using 20′,70′-dichlorodihydrofluorescein diacetate (DCHFDA) as described by Nasr-Esfahani et al [62]. Stock solution of DCHFDA was prepared in dimethyl sulfoxide at 1 mM and stored in the dark at −20°C. Immediately before use, the stock solution was diluted to 0.01 mM in M2. Oocytes were stained for 15 min with the DCHFDA solution. After being washed thoroughly to remove the traces of the dye, 10 oocytes were placed on a slide, covered with a coverslip, and observed under a Leica laser scanning confocal microscope. The fluorescence was obtained by excitation at 488 nm. Photographs were taken using fixed microscopic parameters, and the fluorescence intensity from each oocyte was analyzed using Leica software.

### Data analysis

There were at least three replicates for each treatment. Percentage data were arc-sine transformed and analyzed with ANOVA; a test of Duncan multiple comparisons was used to locate differences. The software used was Statistics Package for Social Science (SPSS 11.5; SPSS Inc., Chicago, IL, USA). Data were expressed as mean ± S.E.M. and P<0.05 was considered significant.
